# Effects of physical activity on visuospatial working memory in healthy individuals: A systematic review and meta-analysis

**DOI:** 10.3389/fpsyg.2023.1103003

**Published:** 2023-02-15

**Authors:** Qiqi Zhu, Jie Deng, Meixi Yao, Chong Xu, Demin Liu, Liya Guo, Yu Zhu

**Affiliations:** ^1^College of Physical Education, Southwest University, Chongqing, China; ^2^Physical Education College, Zhengzhou University, Henan, China; ^3^Ministry of Sports and National Defense Education, Chongqing College of Electronic Engineering, Chongqing, China

**Keywords:** physical activity, visuospatial working memory, healthy individuals, children, seniors

## Abstract

**Introduction:**

Physical activity interventions improve cognitive performance, especially visuospatial working memory (VSWM). However, evidence on the effects of these interventions in children, adolescents, and older adults remains scant. This meta-analysis aimed to identify the effects of physical activity on VSWM improvement in healthy individuals and the best exercise intervention program to improve VSWM capacity.

**Methods:**

We searched for randomized controlled trials (RCTs) of exercise interventions targeting VSWM in healthy individuals from Web of Science, MEDLINE, BIOSIS Previews, PubMed, China National Knowledge Infrastructure, and Wanfang Data (Chinese) databases, from inception to August 20, 2022.

**Results:**

Among 21 articles (1,595 healthy participants), the heterogeneity test statistic was I2 = 32.3%, p = 0.053. The mean quality scores of the included articles were 6.9 points (reaction time [RT] studies) and 7.5 points (Score studies). Moreover, 28 RCTs were included (10 RT studies and 18 Score studies), and the subgroup analysis found significant effects for elderly participants, children, interventions involving a higher level of cognitive engagement, low and moderate exercise intensity, chronic exercise, exercise duration ≥60 min, and exercise period ≥90 days. Physical activity had a small but significant positive impact on VSWM in healthy individuals. Current evidence confirms the effects of physical activity on VSWM capacity only in children and seniors but not in young adults. Other age groups, including adolescents and middle-aged adults, have not been studied. Prescription of interventions involving high-level cognitive engagement, low and moderate exercise intensity, chronic exercise, exercise for >30 min per session, and exercise for more than 3 months is recommended for children and seniors.

**Discussion:**

Future RCTs would be to fill the gap in studies on adolescents and middle-aged adults, and report detailed exercise intervention programs about different age groups.

**Systematic Review Registration:** PROSPERO (https://www.crd.york.ac.uk/prospero/display_record.php?ID=CRD42022354737). INPLASY (https://doi.org/10.37766/inplasy2022.8.0053).

## 1. Introduction

The concept of working memory (WM) evolved in 1974 from the concept of short-term memory; the former emphasizes the ability to manipulate short-term information, whereas the latter emphasizes storage of messages (Baddeley and Hitch, 1974). WM is conceptualized as the ability to mentally retain and manipulate information (Mesulam, 2000). The multi-component model (Baddeley, 2003) clearly explains the composition and specific meaning of WM; it emphasizes storage and manipulation of information and comprises four mutually independent modules. The phonological loop is primarily responsible for storing and processing auditory and linguistic representations. The visuospatial sketchpad is primarily responsible for storing and processing visual and spatial representations. The central executive is responsible for the operation of the entire WM system and supports the interaction between other modules, as well as the interaction between WM and long-term memory. The episodic buffer is responsible for storing the binding representations between various types of information, both internal and external to the module.

Visuospatial WM (VSWM) is a relatively common name for the visuospatial sketchpad, consisting of visual WM (VWM) and spatial WM (SWM), with the two WM components being both independent and interconnected (Baddeley, 2001). Although VWM and SWM emphasize the non-semantic information of the “what” and “where” of the identified object, respectively, they are still complementary in many cases (Baddeley, 2003). Individuals cannot live, learn, or work efficiently without using VSWM. Its deficiency or impairment can prevent people from living a normal life, and its degeneration can lead to inefficiency in learning and work. Therefore, VSWM has been the subject of research since the 1970s.

Visuospatial WM in children and adolescents is of particular importance in academic performance and mental health. Children’s VSWM is associated with school success. For example, the reading process involves encoding of visual and spatial information (Fletcher-Flinn and Thompson, 2007), and a positive correlation exists between visual processing efficiency and reading accuracy (Ferretti et al., 2008). Mathematics learning involves visuospatial and visual perceptual abilities, and deficits in these abilities are likely to contribute to children’s mathematical difficulties (Rasmussen and Bisanz, 2005). Furthermore, handwriting has an important influence on academic performance, and children’s visual information processing and visuomotor integration abilities are related to their handwriting abilities, particularly visual non-motor processing (visual sequential memory and visual closure) and visuomotor integration (Feder et al., 2005; Feder and Majnemer, 2007). In addition, one study confirmed that VSWM is strongly correlated with performance in subjects such as mathematics, English, and science (St Clair-Thompson and Gathercole, 2006). However, deficits in VSWM can affect children’s social interaction processes and even lead to social dysfunction (Kofler et al., 2018), which can negatively affect mental health (Mueller et al., 2015).

Both children’s and adults’ academic performance is linked to VSWM measures, especially in mathematics (Furst and Hitch, 2000) and reading comprehension (Pham and Hasson, 2014). Basic skills related to mathematics and reading comprehension are required in many occupations, and more so in fields such as architecture and engineering (Verstijnen et al., 1998). Hence, adults’ VSWM competencies are tied to their career development.

Visuospatial WM is vital for older people to enjoy a normal old age; however, it has a definite tendency to degenerate with age, with a progressively increased risk for developing dementia, Alzheimer’s disease (AD), and other neurodegenerative diseases closely related to WM (Bo et al., 2009; Leung et al., 2015). Fortunately, exercise seems to slow down this degenerative process (Tsai et al., 2019). Many studies confirm the preventive effect of physical activity on the development of AD—either sporadic AD (Maliszewska-Cyna et al., 2017) or genetically influenced AD (Smith et al., 2011)—in older people and that even light exercise can reduce the risk for the disease. Physical activity is negatively associated with the risk for developing AD. The fact that physically active healthy older adults are less likely to develop AD than those who are sedentary or less physically active is widely accepted; specifically, they have better executive functions, such as WM.

Previous studies have explored various neurophysiological mechanisms *via* which exercise can improve WM and thus prevent AD in older adults. For example, exercise promotes an increase in gray matter in the hippocampus and other brain structures, and this increase is positively correlated with the amount of exercise (Erickson et al., 2010); furthermore, exercise increases the amount of *N*-acetylaspartate, a marker of neuronal activity that decreases with age, thereby increasing neuronal activity (Erickson et al., 2012) and improving WM (Xi et al., 2011). More importantly, brain-derived neurotrophic factor (BDNF) is associated with WM as a neurotrophin involved in regulating dendritic and synaptic plasticity in the hippocampus, and interestingly, it can be induced by doing exercise. Experimental studies in rats have shown that exercise can significantly increase BDNF expression in hippocampus compared to control groups (Sable et al., 2021). Activity-regulated cytoskeleton-associated protein (ARC) plays a vital role in the AMPARs trafficking, which is another crucial biochemical marker that can also be induced through exercise (Garcia et al., 2017).

The improvement effect of exercise on VSWM has been confirmed by many studies, especially the significant positive effects on patients with mild cognitive impairment (Law et al., 2013), dementia (Cheng et al., 2014), schizophrenia (Fujii et al., 2020), and depression (Greer et al., 2015; Brondino et al., 2017), as well as on children with attention deficit hyperactivity disorder (ADHD) (Bustamante et al., 2016; Benzing et al., 2018). Correspondingly, positive effects have been found for exercise programs such as aerobic exercise (Tsai et al., 2014), tai chi (Liao et al., 2021), and open-skill exercise (Guo et al., 2016). However, improvement of VSWM ability is also necessary for healthy people. More importantly, whether these exercise programs can be transferred or extended to healthy individuals, and whether there are exercise programs suitable for healthy people of different ages and sexes, needs to be further analyzed.

A previous meta-analysis explored the effects of exercise in three main areas: (1) non-healthy populations experiencing various cognitive-related disorders or impairments (AD, depression, ADHD, etc.); (2) exercise programs such as aerobic exercise, mind–body exercise (combination of slow physical activity, abdominal breathing, and meditation; for example, tai chi, yoga, and Qigong; Wei et al., 2020; Xiong et al., 2021), open-skill exercise (Zhu et al., 2020), coordinative exercise (Ludyga et al., 2022), artistic gymnastics (Serra et al., 2021), and acute moderate-intensity exercise (McMorris et al., 2011); and (3) studies on the superordinate concepts of VSWM: WM, cognitive function, executive function, and other concepts. However, for cognitively healthy people, few studies exist on the intervention effect of exercise on VSWM, and no consistent results have been obtained. If an individual needs to increase exercise to improve VSWM or delay VSWM decline with age in the absence of impairment or abnormal decline in cognitive ability (unlike decline with age), most effective exercise program, the optimal duration of each exercise, and the shortest effective period of exercise need to be further explored. Furthermore, whether the program is available for healthy people of all ages and sexes also needs to be further discussed. Therefore, a necessity has arisen to conduct a systematic, comprehensive, objective, and quantitative review to further summarize and refine a specific exercise prescription for healthy people and explore whether the improvement and enhancement effects on VSWM are significant.

Thus, this meta-analysis makes innovative contributions to the improvement of VSWM through exercise concerning individual characteristics, qualitative characteristics of exercise, and quantitative characteristics of exercise. Results of this meta-analysis will help the development of more targeted exercise prescriptions, identify the knowledge gap between researchers and practitioners, and provide evidence-based recommendations to clinicians to refine prescriptions for the prevention of VSWM capabilities degradation.

## 2. Methods

The protocol for this systematic review was registered on PROSPERO (CRD42022354737) and INPLASY (INPLASY202280053) and is available in full on inplasy.com (10.37766/inplasy2022.8.0053). The meta-analysis process covered the requirements of the 27-item checklist of the Preferred Reporting Items for Systematic Reviews and Meta-Analyses statement (Liberati et al., 2009). The analysis methods and inclusion criteria were specified in advance and documented in the protocol.

### 2.1. Eligibility criteria

Studies were selected by two authors (QZ and JD) according to the following inclusion criteria: (1) participants were of any age and sex and identified as cognitively and physically healthy *via* validated diagnostic tools; (2) participants had normal or corrected visual acuity; (3) intervention measures were all kinds of physical activities (two conditions must be met: skeletal muscle movement and energy expenditure); (4) all outcomes were quantified by validated VSWM measuring tools; and (5) the study was a randomized controlled trial (RCT) as RCTs have the highest quality of evidence in meta-analysis. Studies meeting the following criteria were excluded: (1) control measures include physical activity; (2) participants had concussions or other brain injuries; or (3) participants had contraindications to exercise or were taking medication. Reports meeting the following criteria were also excluded: (1) language of publication was not English or Chinese and (2) full text or important data (i.e., mean and standard deviation) were not available.

### 2.2. Information sources

We searched six electronic databases [Web of Science, MEDLINE, BIOSIS Previews, PubMed, China National Knowledge Infrastructure, and Wanfang Data (Chinese)] from inception to August 20, 2022. These databases contained almost all the potential RCTs we needed. Because of the limited human resources available for this review, only these six databases were selected.

### 2.3. Search strategy

A systematic search strategy was applied using MeSH word search. For example, the following retrieval strategy was used: “Exercise” [MeSH] (e.g., “Exercises” OR “Physical Activity” OR “Activities, Physical” OR “Activity, Physical” OR “Physical Activities” OR “Exercise, Physical” OR “Exercises, Physical” OR “Physical Exercise” OR “Physical Exercises” OR “Acute Exercise” OR “Acute Exercises” OR “Exercise, Acute OR Exercises, Acute” OR “Exercise, Isometric” OR “Exercises, Isometric” OR “Isometric Exercises” OR “Isometric Exercise” OR “Exercise, Aerobic” OR “Aerobic Exercise” OR “Aerobic Exercises” OR “Exercises, Aerobic” OR “Exercise Training” OR “Exercise Trainings” OR “Training, Exercise” OR “Trainings, Exercise”) AND “Memory, Short-Term” [MeSH] (e.g., “Memories, Short-Term” OR “Memory, Short Term” OR “Short-Term Memories” OR “Short-Term Memory” OR “Memory, Short term” OR “Memories, Short term” OR “Short term Memories” OR “Short term Memory” OR “Working Memory” OR “Working Memories” OR “Memory, Immediate” OR “Immediate Memories” OR “Immediate Memory” OR “Memories, Immediate” OR “Immediate Recall” OR “Immediate Recalls” OR “Recall, Immediate” OR “Recalls, Immediate”).

### 2.4. Data extraction and coding

All documents obtained through the search strategy were imported into EndNote document management software to eliminate duplicates. The eligibility assessment was performed independently in an unblinded standardized manner by two authors (QZ and JD). Disagreements between reviewers were resolved by consensus. Information was extracted using a pre-designed data extraction form that included (1) basic information: author and year of publication; (2) participant characteristics: average age or age range in the experimental and control groups separately, and sample size; (3) experimental characteristics: study design, intervention type, control type, intervention information (intervention duration, intervention session time, intervention frequency, intervention intensity), measurement tools, and outcome indicators. One author (QZ) extracted the following data from the included studies, and the second author (JD) checked the extracted data. Disagreements were resolved through consensus. If agreement could not be reached, the third author (YZ) decided.

### 2.5. Study risk of bias assessment

Two authors (QZ and JD) independently assessed the risk for bias of the included studies according to the Physiotherapy Evidence Database (PEDro) scale (Maher et al., 2003), which comprises the following 11 items: eligibility criteria, randomization, concealed allocation, similar baseline, blinding of participants, blinding of therapists, blinding of assessors, more than 85% retention, intent-to-treat analysis, between-group comparison, and point measures and measures of variability. The total score of the quality evaluation was calculated from the scores of 10 items, excluding the first item (eligibility criteria). The range of the total score was 0–10; a score ≥ 6 indicated high quality of the assessed studies, and a score < 6 indicated low quality (Maher et al., 2003). Any disagreements between the reviewers were resolved through consultation with another reviewer (YZ).

### 2.6. Statistical syntheses and analysis

The meta-analyses were performed by computing standardized mean differences (SMDs) using a random-effects model in Stata 15.1 software. SMDs and 95% confidence intervals (CI) for each study were calculated. The primary outcome measure was the standardized mean difference in the VSWM-related tests or scale results of the exercise intervention and control groups. Heterogeneity was calculated using *I*^2^ statistic with 95% CI, with 0, 25, 50, and 75% as the thresholds for none, low, medium, and high ratios of the included studies, respectively. Sources of heterogeneity were analyzed through subgroup analysis, meta-regression analysis, or re-run meta-analysis, by excluding studies with abnormal results.

Studies that met the inclusion and exclusion criteria were critically reviewed, and intervention characteristics were extracted for tabulation. VSWM scores and RT were the main outcome indicators. SMD was used for the combined effect sizes separately and was calculated using Hedges’ *g*. The resulting effect sizes were transformed to bias-corrected Hedges’ *g*, with values of 0.2, 0.5, and 0.8, which served as thresholds for small, medium, and large effects, respectively. Forest plots of the pooled effect sizes were drawn separately from the Score and RT data. The size of heterogeneity was tested using forest plots, and *I*^2^ and its 95% CI were reported. If *I*^2^ was >50% (*p* < 0.1), the source of heterogeneity was further analyzed using subgroup analysis and meta-regression analysis. If no source of heterogeneity was found, the random-effects model was selected directly for statistical analysis. If no heterogeneity was detected, a fixed-effects or random-effects model was directly selected for statistical analysis.

Subgroup analyses were conducted concerning six moderators: (1) Age was divided into three subgroups: children, young adults, and seniors; improvements in VSWM may vary with age and may be more significant during childhood and old age, which are two periods of rapid change—development and aging (Kupis et al., 2021). (2) Level of cognitive engagement was divided into two subgroups: high level and low level; exercise interventions with different cognitive requirements may have different improvement effects on VSWM (Formenti et al., 2021). (3) Exercise intensity was divided into two subgroups: low and moderate intensity and vigorous intensity; whether the intensity of exercise intervention will affect the improvement effect of exercise on VSWM is of significance for the clinical improvement of healthy people (Loprinzi et al., 2022). (4) Intervention time was divided into two subgroups: acute exercise and chronic exercise; acute exercise is defined as one-off exercise of relatively short duration, while chronic exercise is defined as exercise that lasts for a long time, several times a week, for weeks or years; a review showed that both acute and chronic exercise can improve the performance of memory systems (Loprinzi et al., 2021); however, the two may have different improvement effects on VSWM; therefore, further discussion is needed. (5) Intervention period was divided into three subgroups: <30, 30–89, and ≥90 days. Does a longer exercise cycle have a better effect on VSWM? Is there a peak for effect size? These questions require further subgroup analysis. (6) Intervention duration was divided into three subgroups: <30, 30–59, and ≥60 min; previous experiments in rats showed that increased exercise duration affects the level of WM enhancement (Sinaei et al., 2021), but whether the same results apply to healthy humans is open to further discussion.

### 2.7. Risk for publication bias assessment

We created a funnel plot to assess publication bias using the standard error and its inverse for each included study. The symmetry of the funnel plot indicated no risk for publication bias, both visually and formally, using the Egger’s test. Because graphical evaluation can be subjective, we also conducted an adjusted rank correlation test (Begg test) as another formal statistical test to confirm whether publication bias exists.

## 3. Results

### 3.1. Literature search results

**Figure 1:**
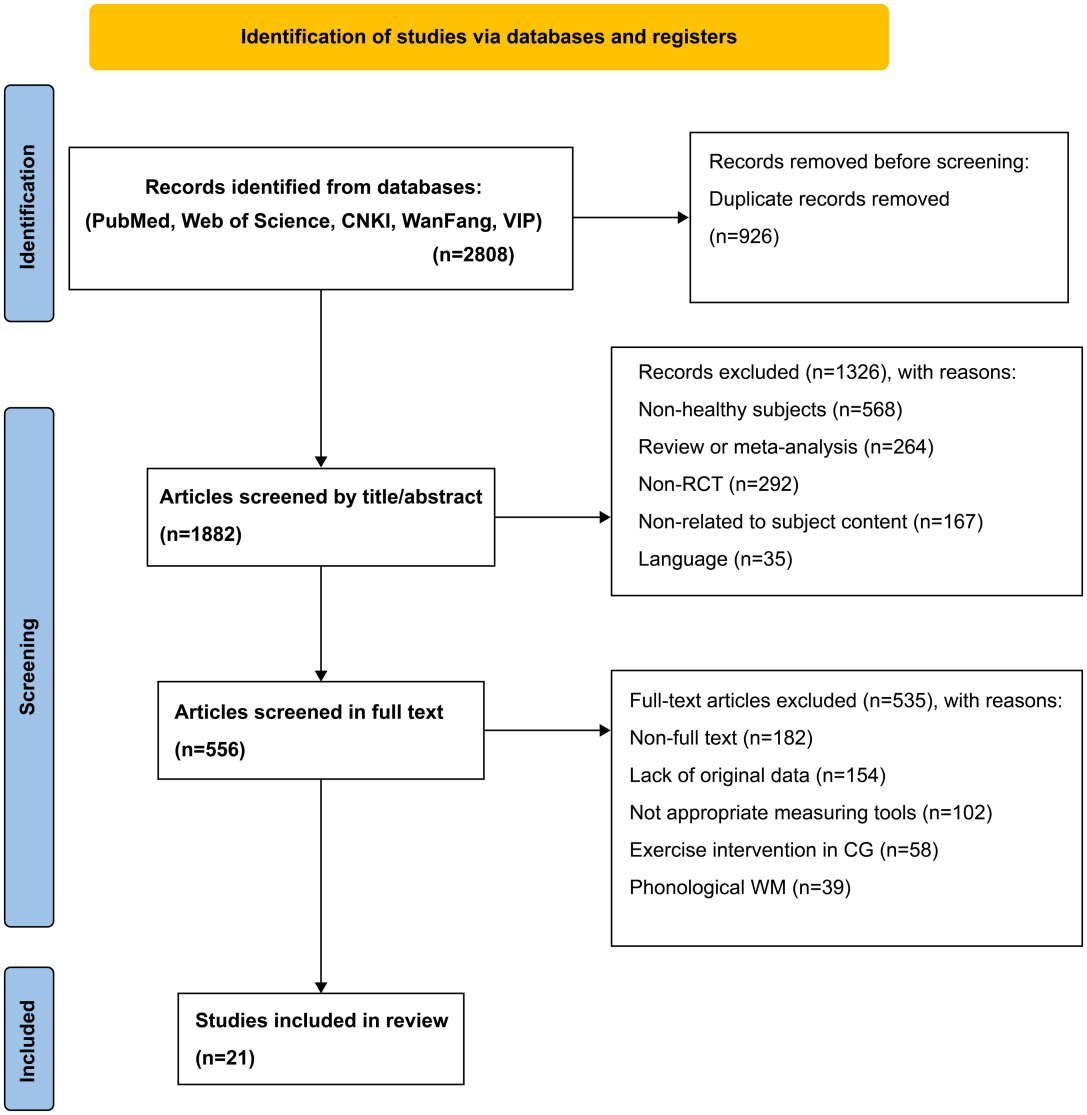
Flow of the study selection method.

Initially, 2,808 articles were searched from six databases. All articles were imported into EndNote X9, and 926 duplicates were removed. In the preliminary review phase, 1,326 articles were excluded after reading the titles and abstracts; reasons for exclusion were having non-healthy participants (*n* = 568), being reviews or meta-analyses (*n* = 264), being non-RCT (*n* = 292), being unrelated to subject content (*n* = 167), and being non-Chinese or non-English (*n* = 35). Another 535 articles were excluded after reading the full text, and reasons for exclusion included non-full text (*n* = 182), lack of original data (*n* = 154), inappropriate measuring tools (*n* = 102), exercise intervention in the control group (*n* = 58), and phonological WM (*n* = 39). Finally, 21 articles were included in the review (Figure 1).

### 3.2. Eligible research characteristics

After review, 21 articles, including 28 RCTs, were included. The average intervention period was 10.9 weeks, the average intervention duration was 41.3 min, and the main intervention was aerobic exercise. RT and Score were the main outcome indicators in these articles. Among them, the visual N-back task and N-back (1-back, 2-back) measurement tools were mainly used in RT studies. In addition, measurement tools such as Digit Span Backwards (DSB), Digit Span Forward (DSF), Digit Span (DS), Visual Paired Associates (VISPA), VSWM, and spatial attention tasks were used in Score studies. The sample size comprised 1,595 healthy participants, who were divided into three age groups: children (4–11 years), young adults (18–35 years), and seniors (58–88 years). Except for one study that did not report sex proportion, 27 studies reported a female participation rate of 80.9% (Table 1).

**Table 1:** Characteristics of the studies included in the meta-analysis.

Reference (year)	Study design	Individual characteristic	Group	Intervention type	Control type	Intervention characteristic	Measuring tool	Outcomes measurement
Li (2019)	RCT	Community seniors, *n* = 74 EG age: 66.66 ± 4.89 CG age: 65.97 ± 4.13	EG: *n* = 38 CG: *n* = 36	Five mimic-animal exercises	NM	40 weeks 1 session/week 60 min/session	③⑤⑥	RT
Peng and Zhou (2016)	RCT	Female university students, *n* = 59 EG age: 21.33 ± 1.49 CG age: 21.0 ± 0.78	EG: *n* = 31 CG: *n* = 28	Acute aerobic exercise (Power cycling)	R	30 min	③	RT
Yang et al. (2019)	RCT	Women seniors, *n* = 52 EG-a age: 64.5 ± 3.9 EG-b age: 62.6 ± 4.6 CG age: 63.2 ± 5.3	EG-a: *n* = 16 EG-b: *n* = 19 CG: *n* = 17	Taijiquan; Square dance	W	6 months ≥5 sessions/week 75 min/session	③	RT
Zhang H. (2021)	RCT	University students, *n* = 56 EG age: 18.53 ± 0.57 CG age: 18.46 ± 0.71	EG: *n* = 30 CG: *n* = 26	Medium-intensity aerobic exercise	NM	9 weeks 4 sessions/week 60 min/session	⑦	RT
Lindheimer et al. (2017)	RCT	Healthy adults, *n* = 60	EG-a: *n* = 15 EG-b: *n* = 15 CG-a: *n* = 15 CG-b: *n* = 15	Cycling	PC	30 min/session	⑧	RT
Nishiguchi et al. (2015)	RCT	Community-dwelling older adults, *n* = 48 EG age: 73.0 ± 4.8 CG age: 73.5 ± 5.6	EG: *n* = 24 CG: *n* = 24	Dual task–based multimodel exercise; Walking exercise	NM	12 weeks 90 min/session	③	RT
Li et al. (2020)	RCT	Female university students, *n* = 60 EG-a age: 18.44 ± 0.51 EG-b age: 18.43 ± 0.51 CG age: 18.58 ± 0.51	EG-a: *n* = 18 EG-b: *n* = 21 CG: *n* = 19	Aerobic exercise; Resistance exercise	NM	8 weeks 3 sessions/week 60 min/session	③	RT
Wu (2021)	RCT	Children, *n* = 40 Range age: 4–5	EG: *n* = 20 CG: *n* = 20	Aerobic gymnastics	DR	8 weeks 2 sessions/week 60 min/session	⑫	RT
Brown et al. (2009)	RCT	Seniors, *n* = 154 EG-a age: 79.5 ± 5.9 EG-b age: 81.5 ± 6.9 CG age: 78.1 ± 6.4	EG-a: *n* = 66 EG-b: *n* = 26 CG: *n* = 34	Flexibility and relaxation; Specific resistance training; Balance (both static and dynamic) training	NM	6 months 2 sessions/week 60 min/session	①②④⑨	Score
Elkana et al. (2018)	RCT	Adults, *n* = 69 EG age: 25.43 (range: 19–34) CG age: 26.39 (range: 22–35)	EG: *n* = 35 CG: *n* = 34	Acute physical exercise	NM	15 min	⑩	Score
Moreau et al. (2017)	RCT	Children, age: NR	EG: *n* = 152 CG: *n* = 153	HIIT	NM	6 weeks	①	Score
Wilke (2020)	RCT	College students with exercise background, *n* = 35 EG-a age: 26.5 ± 4.4 EG-b age: 26.4 ± 2.6 CG age: 27.3 ± 3.8	EG-a: *n* = 12 EG-b: *n* = 11 CG: *n* = 12	HIIT; Aerobic walking	R	15 min	①②④	Score
Kramer et al. (2002)	RCT	Sedentary older adults, *n* = 125 EG age: 67.3 ± 5.2 CG age: 66 ± 5.3	EG: *n* = 66 CG: *n* = 58	Aerobic exercise	S	6 months 3 sessions/week 40 min/session	①②⑪	Score
Nouchi et al. (2014)	RCT	Seniors, *n* = 64 EG age: 66.75 ± 7.61 CG age: 67.06 ± 2.82	EG: *n* = 32 CG: *n* = 32	Aerobic; Strength; Stretching	NM	4 weeks 3 sessions/week	①②	Score
Tottori et al. (2019)	RCT	Children, *n* = 56 EG age: 10.0 ± 1.0 CG age: 10.4 ± 1.1	EG: *n* = 27 CG: *n* = 29	HIIT	NM	4 weeks 3 sessions/week 8–10 min/session	①②	Score
Kalbe et al. (2018)	RCT	Seniors, *n* = 55 EG-a age: 68.22 ± 7.96 EG-b age: 68.75 ± 6.62 CG age: 67.53 ± 5.89	EG-a: *n* = 18 EG-b: *n* = 20 CG: *n* = 17	Cognitive and physical training	CT	7 weeks 2 sessions/week 90 min/session	①	Score
Hong et al. (2018)	RCT	Seniors, *n* = 25 EG age: 76.50 + ±6.36 CG age: 73.50 ± 5.57	EG: *n* = 12 CG: *n* = 13	Resistance exercises	NM	12 weeks 2 sessions/week 60 min/session	①②	Score
Hariprasad et al. (2013)	RCT	Seniors, *n* = 120 EG age: 75.74 ± 6.46 CG age: 74.78 ± 7.35	EG: *n* = 62 CG: *n* = 58	Yoga	NM	6 months 3–4 sessions/week 60 min/session	①②⑬⑭	Score
Fabre et al. (2002)	RCT	Seniors, *n* = 24 EG-a age: 65.4 ± 2.2 EG-b age: 64.9 ± 1.4 CG age: 65.7 ± 1.5	EG-a: *n* = 8 EG-b: *n* = 8 CG: *n* = 8	Aerobic exercise; Mental training	NM	2 months 2 sessions/week 60 min/session	②	Score
Li (2016)	RCT	Seniors, *n* = 66 EG-a age: 67.35 ± 4.29 EG-b age: 66.59 ± 4.02 CG age: 65.93 ± 5.13	EG-a: *n* = 22 EG-b: *n* = 25 CG: *n* = 19	Aerobic walk; Baduanjin	NM	6 months 3 sessions/week 60 min/session	⑮	Score
Zhang B. (2021)	RCT	Children, *n* = 48 EG age: 11.167 ± 0.38 CG age: 11.208 ± 0.41	EG: *n* = 24 CG: *n* = 24	Rhythmic exercise	DR	18 weeks 3 sessions/week 40 min/session	⑯	Score

NR, not reported; EG-a, Experimental group a; EG-b, Experimental group b; CG-a, Control group a; CG-b, Control group b; RCT, randomized controlled trial; NM, no movement; CT, cognitive training; DR, daily routine; S, stretching; R, reading; PC, passive cycling; W, Walk; ① DSB, Digits Span Backward; ② DSF, Digits Span Forward; ③ N-back; ④ DS, Digits span; ⑤ Stroop; ⑥ ANT, Attention Network Test; ⑦ 2-back; ⑧ Visual N-back Task; ⑨ VISPA, Visual Paired Associates; ⑩ VS-WM, Visuospatial Working Memory; ⑪ SAT, Spatial Attention Task; ⑫ 1-back; ⑬ SSB, Spatial Span Backward; ⑭ SSF, Spatial Span Forward; ⑮ WMS, Wechsler Memory Scale; ⑯ SWMs, Spatial Working Memory score.

### 3.3. Methodological quality evaluation

The quality of the 21 included articles was evaluated using the PEDro Scale (Table 2). The mean score for the quality of the RT articles was 6.9 points and that for the Score articles was 7.6 points. In addition, all articles reported “statistical analyses between groups” and “point measures and measures of variance” and used random allocation for experimental grouping. Moreover, 12 articles described the process of random allocation, 18 reported on baseline levels of participants, and 18 provided key outcome measures for over 85% of participants. Eighteen articles reported a retention rate of ≥85% and completeness of the measurement results.

**Table 2:** Physiotherapy evidence database (PEDro) scores and sum of the included studies.

Articles	Item 1	Item 2	Item 3	Item 4	Item 5	Item 6	Item 7	Item 8	Item 9	Item 10	Item 11	Sum (Items 2–11)
Reaction time (RT)												
Li (2019)	1	1	0	1	1	1	0	1	1	1	1	8
Peng and Zhou (2016)	1	1	0	1	0	0	0	1	1	1	1	6
Yang et al. (2019)	1	1	0	1	0	0	0	1	1	1	1	6
Zhang H. (2021)	1	1	0	1	0	0	0	1	1	1	1	6
Lindheimer et al. (2017)	1	1	0	1	1	0	0	1	1	1	1	7
Nishiguchi et al. (2015)	1	1	1	1	1	1	0	1	1	1	1	9
Li et al. (2020)	1	1	0	1	0	0	0	1	1	1	1	6
Wu (2021)	1	1	1	1	0	0	0	1	1	1	1	7
Mean (RT)												6.9
Scores												
Brown et al. (2009)	1	1	1	1	1	0	0	1	1	1	1	8
Elkana et al. (2018)	1	1	1	1	0	0	0	0	1	1	1	6
Moreau et al. (2017)	1	1	1	1	1	0	1	1	1	1	1	9
Wilke (2020)	1	1	1	1	1	0	0	1	1	1	1	8
Kramer et al. (2002)	1	1	1	1	1	0	0	1	1	1	1	8
Nouchi et al. (2014)	1	1	1	1	0	0	1	1	1	1	1	8
Tottori et al. (2019)	1	1	0	1	0	0	0	1	1	1	1	6
Kalbe et al. (2018)	1	1	0	1	0	0	0	1	1	1	1	6
Hong et al. (2018)	1	1	0	1	1	0	0	1	1	1	1	7
Hariprasad et al. (2013)	1	1	1	1	1	0	0	1	1	1	1	8
Fabre et al. (2002)	1	1	1	1	1	0	0	1	1	1	1	8
Li (2016)	1	1	1	1	1	1	1	1	1	1	1	10
Zhang B. (2021)	1	1	1	0	0	0	0	1	1	1	1	6
Mean (Score)												7.5

Item 1: eligibility criteria; Item 2: randomization; Item 3: concealed allocation; Item 4: similar baseline; Item 5: blinding of participants; Item 6: blinding of therapists; Item 7: blinding of assessors; Item 8: more than 85% retention; Item 9: intent-to-treat analysis; Item 10: between-group comparison; Item 11: point measures and measures of variability.

### 3.4. Meta-analysis results

#### 3.4.1. Heterogeneity test

A meta-analysis was conducted on 28 experiments, which were included in 21 articles on exercise interventions in VSWM. Among these, 10 were RT studies and 18 were Score studies. According to the heterogeneity test, the meta-analysis results of the 21 combined articles were as follows: *I*^2^ = 32.3%, *p* = 0.053; mild heterogeneity was observed. The heterogeneity of the RT-related studies was *I*^2^ = 0, *p* = 0.677 and that of the Score studies was *I*^2^ = 0, *p* = 0.897. Heterogeneity results show that the selection of outcome indicators may be the source of heterogeneity; therefore, the two types of indicators were analyzed separately (Table 3).

**Table 3:** Heterogeneity analysis of exercise effects of included studies.

	Heterogeneity		Effect size			
	*I*^2^ (%)	*P*	SMD	95% Cl	Z	*P*
RT	0	0.677	−0.202	[−0.393, −0.012]	2.08	0.037
Score	0	0.897	0.342	[0.228, 0.457]	5.86	0.000
Total	32.3	0.053	0.198	[0.100,0.296]	3.95	0.000

#### 3.4.2. Overall effect size

**Figure 2:**
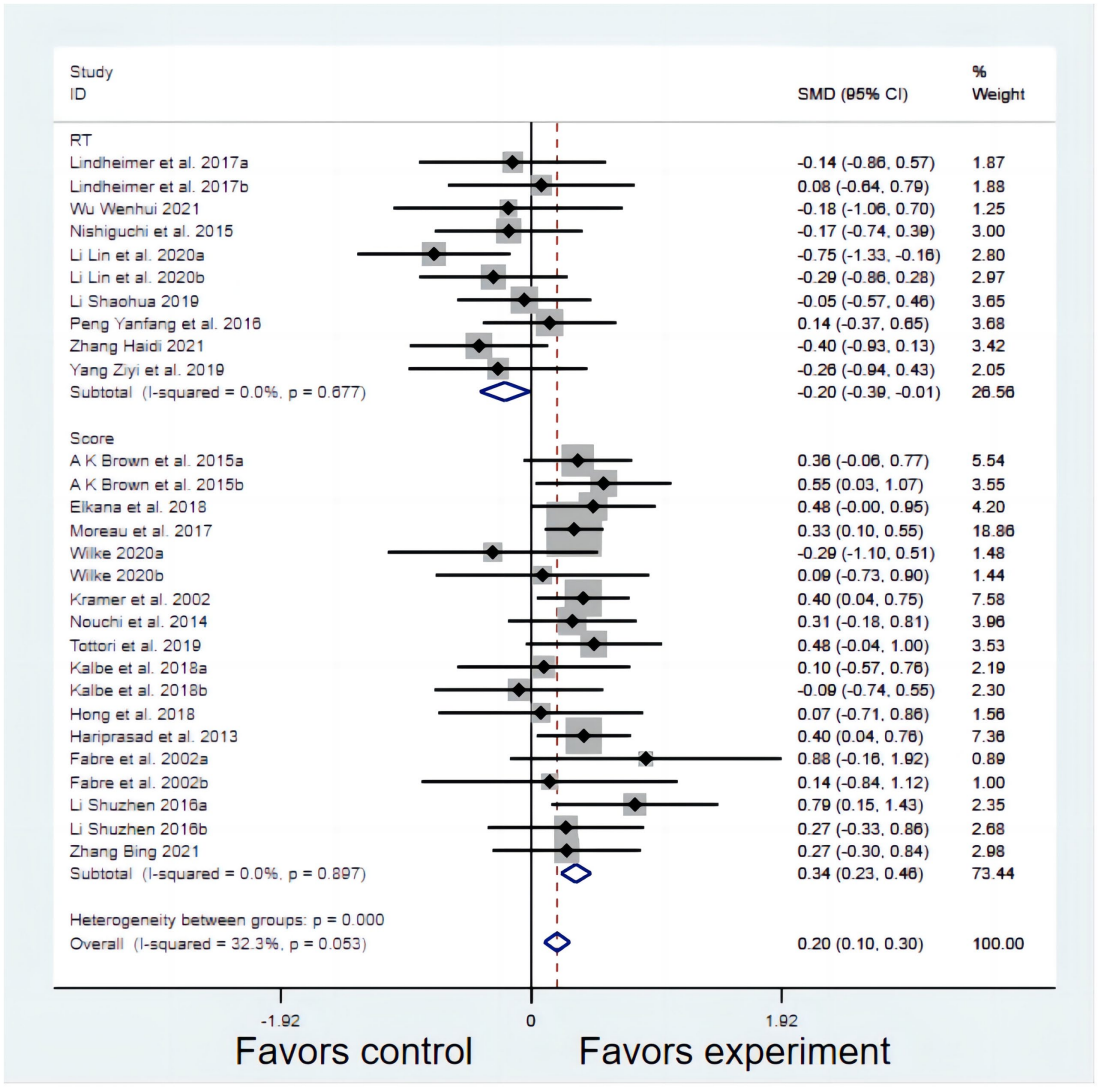
The effect of reaction time (RT) and score.

Twenty-eight studies investigated the effects of exercise interventions on VSWM (Figure 2). According to the heterogeneity test, the choice of the outcome index would affect the statistical results. Therefore, RT and Score are discussed separately in these studies, and the effect size was calculated separately for the RT and Score studies. The effect size of Score studies was SMD = 0.342, *p* = 0.000, which was significant. The effect size of RT studies was SMD = −0.202, *p = 0*.037, which was also significant (Table 3).

#### 3.4.3. Publication bias results

**Figure 3:**
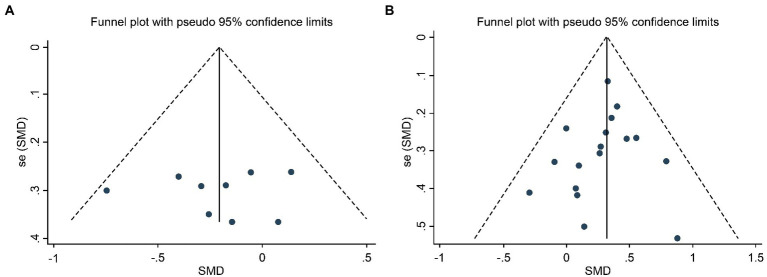
Funnel Plot of Publication Bias of RT Studies **(A)** and Score Studies **(B)**.

We tested the included articles for publication bias, and the results showed that the RT (Figure 3A) and Score (Figure 3B) studies were evenly distributed in the funnel and approximately symmetrically distributed. Therefore, we preliminarily determined that these studies had no publication bias. To confirm the reliability of the results, Begg and Egger’s tests were used to further evaluate publication bias objectively, which showed that RT (*p* = 0.954, 95% CI: −4.300–4.085) and Score (*p* = 0.264, 95% CI: −0.400–0.117) studies were free of publication bias.

### 3.5. Results of subgroup analysis

The overall forest map shows that physical exercise can effectively improve the VSWM ability of healthy individuals. The reasons for not using RT studies for subgroup analysis are as follows: the quality of RT studies is low and the weight is small. For the exercise interventions, variables (age, level of cognitive engagement, exercise intensity, intervention times, intervention period, and intervention duration) were likely to be influencing factors for healthy participants on VSWM. Consequently, subgroup analysis was used to help identify more appropriate exercise programs.

Participants were divided into three age subgroups: children, young adults, and seniors; the effect of the exercise interventions on VSWM was affected by the participants’ age. Exercise had the best improvement effect on VSWM in senior participants (SMD = 0.361, *p* = 0.000), followed by that in children (SMD = 0.343, *p* = 0.001).

The types of exercise intervention included three groups of factors: level of cognitive engagement, exercise intensity, and intervention times. First, the effect size of the exercise intervention on VSWM was affected by the level of cognitive engagement: exercise interventions with a higher level of cognitive engagement had the best improvement effect on VSWM (SMD = 0.336, *p* = 0.000). Moreover, exercise interventions with low levels of cognitive engagement also partly improved VSWM (SMD = 0.254, *p* = 0.027). Second, the effect size of the exercise intervention on VSWM was affected by exercise intensity, and the effect of low-and moderate-intensity interventions (SMD = 0.366, *p* = 0.000) was better than that of vigorous interventions (SMD = 0.299, *p* = 0.003). Finally, chronic exercise (SMD = 0.335, *p* = 0.000) had a significant intervention effect on VSWM, whereas acute exercise did not have a significant effect (*p* = 0.192), as shown by the results of the subgroup analysis of acute and chronic exercise.

The results of subgroup analysis showed that exercise intervention duration had an impact on the effect size. Significant effect sizes were observed when the exercise duration was 30–59 min (SMD = 0.349, *p* = 0.008) and ≥60 min (SMD = 0.357, *p* = 0.000), whereas the improvement effect on VSWM was not significant when the duration was <30 min. In addition, the exercise intervention period can also have an impact on the intervention effect. The exercise intervention period was divided into three subgroups (<30, 30–89, and ≥90 days) for the analysis. The results showed that when the period was ≥90 days (SMD = 0.383, *p* = 0.000), the effect of the intervention was the best, which was slightly better than the 30–89-day period (SMD = 0.257, *p* = 0.044), while the <30-day effect was not significant (Table 4).

**Table 4:** Result of subgroup analysis.

Moderator	Subgroup	Score					
		SMD	95%CI	*Z*	*Q*	*df*	*P*
Age	Children	0.343	[0.148,0.538]	3.45	0.34	10	0.001
	Young adults	−0.043	[−0.408,0.321]	0.23	2.76	2	0.816
	Seniors	0.361	[0.207,0.514]	4.61	6.68	2	0.000
Level of cognitive engagement	High	0.336	[0.203,0.469]	4.96	2.35	10	0.000
	Low	0.254	[0.029,0.478]	2.21	7.76	5	0.027
Exercise intensity	Low and moderate	0.366	[0.216,0.517]	4.78	6.99	12	0.000
	Vigorous	0.299	[0.104,0.494]	3.01	2.88	3	0.003
Intervention times	Acute exercise	0.274	[−0.138,0.686]	1.30	2.59	1	0.192
	Chronic exercise	0.335	[0.207,0.463]	5.13	7.45	14	0.000
Intervention period	<30 days	−0.043	[−0.40,0.321]	0.23	2.76	2	0.208
	30–89 days	0.257	[0.008,0.507]	2.02	3.75	6	0.044
	≥90 days	0.383	[0.246,0.521]	5.46	2.53	6	0.000
Intervention duration	<30 min	0.256	[0.075,0.436]	2.78	3.32	4	0.005
	30–59 min	0.349	[0.092,0.607]	2.66	0.16	3	0.008
	≥60 min	0.357	[0.176,0.538]	3.87	6.60	8	0.000

(Q-value, heterogeneity statistic; df, degrees of freedom).

### 3.6. Meta-regression analysis

To test the effect of exercise interventions on VSWM in healthy individuals, a meta-regression analysis was conducted on the variables, including age, quality of evidence, type of control group, exercise intensity, exercise intervention duration, intervention period, and type of intervention, using pooled RT and Score studies. The results showed that children (*β =* 0.41, *p* = 0.021), seniors (*β =* 0.336, *p* = 0.013), PEDro score (*β* = 0.108, *p* = 0.031), low intensity (*β* = 0.407, *p* = 0.017), intervention duration (*β* = −0.006, *p* = 0.029), and intervention period (*β* = 0.012, *p* = 0.036) were significantly correlated with VSWM. However, sedentary behavior (*p* = 0.717), daily routine (*p* = 0.645), vigorous exercise (*p* = 0.106), acute exercise (*p* = 0.541), and high-level cognition (*p* = 0.843) were not significantly associated. In the multivariate meta-regression analysis results, tau^2^ = 0 and Adj *R*^2^ = 1, indicating that the selected variables explained the source of heterogeneity between studies. Consequently, we conducted multivariate meta-regression analysis based on a data-driven model that included variables that were significantly correlated with effect size prediction. The results showed that when controlling for other variables, two variables could significantly predict the effect size of exercise intervention on VSWM: old age and duration of exercise intervention (Table 5).

**Table 5:** Results of the meta-regression models investigating potential moderators of the effect of exercise on VSWM.

Model	Covariate	*β*	Lower 95% CI	Upper 95% CI	*P*	*R*^2^ analog	Tau^2^
1	Age					0.14	0.012
	Children	0.41	0.677	0.753	0.021		
	Seniors	0.336	0.084	0.64	0.013		
2	PEDro score	0.108	0.010	0.207	0.031	0.02	0.47
	Control type						
	Sedentary	−0.065	−0.436	0.304	0.717		
	Daily routine	−0.109	−0.59	0.3744	0.645		
3	Exercise intensity					0.017	0.53
	Low	0.407	0.078	0.735	0.017		
	Vigorous	0.272	−0.062	0.607	0.106		
4	Intervention period	0.012	0.0008	0.023	0.036	0.01	0.73
	Intervention duration	−0.006	−0.012	−0.0007	0.029		
5	Acute exercise	0.096	−0.223	0.415	0.541	−0.15	0.044
	High-level cognitive exercise	0.029	−0.278	0.338	0.843		
6	Children	0.28	−0.115	0.668	0.153	1	0
	Seniors	0.617	0.22	1.01	0.004		
	PEDro score	0.001	−0.125	0.128	0.982		
	Low intensity	0.108	−0.218	0.435	0.497		
	Intervention period	−0.000	−0.15	0.13	0.893		
	Intervention duration	−0.01	−0.17	0.003	0.006		

## 4. Discussion

### 4.1. Summary of evidence

Overall, physical activity had a small but significant positive impact on VSWM in healthy individuals, mainly reflected by the accuracy of the test (i.e., scores). The effect size concerning quantity (Score) was large and highly significant. There was no heterogeneity between studies and no publication bias. The quality of evidence was high, and the results of the meta-analysis were credible. Regarding speed (RT), the effect size was small and non-significant. There was mild heterogeneity between studies but no publication bias. The quality of the evidence was slightly below the eligibility standard; however, the results of the meta-analysis were relatively reliable. To the best of our knowledge, this meta-analysis is the first study to explore the prescription of exercise interventions for VSWM, covering healthy people of all ages. It is necessary to further investigate whether other potential moderators influence the effects of exercise intervention on VSWM. The total effect size was slightly higher (0.341 > 0.217) than that in a previous study in which Score was used as an outcome indicator (Ludyga et al., 2020).

Subgroup analysis showed that the variables age, intervention time, and intervention period had selective effects. The intervention effects were significant for children and older adults, and non-significant for young adults, with almost non-existent effect sizes. There are two possible explanations for this. One is that the VSWM capacity in healthy young adults is at its peak, and increasing it significantly is difficult. Age advantage gives young adults better filtering performance in VWM capacity compared with healthy older adults (Jost et al., 2011). Second, these physical activity prescriptions may not be appropriate for healthy young adults to improve their VSWM ability, and the intervention effects may be associated with factors such as exercise intensity, intervention period, and intervention mode.

The finding that chronic exercise had a significant effect, while acute exercise produced non-significant improvement, in VSWM in healthy individuals is consistent with the results of a previous meta-analysis (Rathore and Lom, 2017). The lack of an effect size may be related to the limited number of relevant studies included. However, previous meta-analyses on children showed large and significant effect sizes for acute exercise in improving children’s WM capacity, even exceeding the effect sizes for chronic exercise (Liu et al., 2020). From this perspective, it would be interesting to see whether the intervention effect of acute exercise is related to the age of participants. The effect of the intervention was significant when the duration of the intervention was ≥30 days, whereas the improvement in cognition was not significant when it was <30 days. Evidence from studies relating white matter plasticity to exercise showed that prolonged intervention and practice was required for at least several weeks (Fields, 2015). An intervention duration of <30 days is relatively short, and changes in white matter are not sufficient to cause significant improvements in WM. Therefore, short-term interventions and measurements cannot ascertain the effect of exercise on the improvement in VSWM.

The variables cognitive engagement, exercise intensity, and intervention duration showed general effects in that no between-group differences were observed in their subgroups, and all had significant effect sizes. Relatively high cognitive engagement, low to moderate exercise intensity, and ≥60 min of intervention length had larger effect sizes than the other subgroups. However, the relative superiority should be interpreted with caution because of the small between-group differences in effect sizes, as we currently do not know the pathway of their mechanisms.

Regression analysis showed that age was positively associated with the overall intervention effect in the univariate regression model. The included studies showed a significant enhancement effect of physical activity on VSWM capacity in healthy populations (mainly children and older adults). Data on young adults were excluded because of covariance issues, and research on healthy adolescents and healthy middle-aged adults was scant. From a neuroscience perspective, WM develops comprehensively throughout childhood, which depends, to some extent, on the maturity of the white matter microstructure of the brain and the development of neural connections. Changes in white matter bundles are associated with changes in children’s VSWM ability but not significantly with changes in phonological WM ability (Krogsrud et al., 2018). VSWM changes in healthy older adults are associated with white matter integrity. Mean diffusivity, a measure of white matter integrity, increases with age, and white matter integrity decreases, affecting WM function. However, previous studies have confirmed that the intervention pathway to achieve a positive impact on WM is to improve white matter integrity, and thus WM capacity, by reducing mean diffusivity in the fiber tracts associated with the frontoparietal network (Dziemian et al., 2021).

In addition to age, the low and moderate intensity groups were positively associated with the overall intervention effect. Motor cognition involves the brain region responsible for motor control and the neural integration of movement and cognition (Leisman et al., 2016). Physical fatigue owing to excessive physical activity and cognitive fatigue owing to prolonged intellectual activity share common basal ganglia (Blain et al., 2019). There are also studies that present a neurological perspective based on motor fatigue: top–down (cognitive and physical efforts) and bottom–up (body sensations) processes act in parallel with arousing mechanisms to determine cognitive outcomes (Schmit and Brisswalter, 2020). From this perspective, low-to-moderate-intensity exercise has some neurological benefits concerning cognitive improvement compared with vigorous intensity.

The study’s quality factor had a small but significant influence on the total effect size. Even though the review team screened the literature in strict accordance with the patient/population, intervention, comparison, and outcomes principle and confirmed the absence of significant publication bias, there could still be relatively low-quality studies that did not meet the individual criteria of the quality assessment, thereby affecting the overall intervention effect. The intervention period and duration also had significant but negligible effects on the total effect size. The analysis of moderating factors showed that the effect size of the period and duration was positively correlated with its own value. However, a previous meta-analysis indicated an interaction between intervention period and duration (Ludyga et al., 2020). Thus, a longer single intervention would be more effective with a minimum of a 30-day intervention.

The exercise interventions identified in the study were specifically targeted at VSWM in healthy individuals, while other exercise interventions were mostly targeted at WM, executive function, or other cognitive abilities in an unhealthy population. Therefore, independent exercise programs can be provided to healthy individuals who want to enhance or improve their VSWM performance and reduce the risk for associated cognitive decline or impairment. In addition, it also provides a new way of thinking and direction for clinical treatment of VSWM-related diseases.

Participants in different RCTs have different initial cognitive levels, which could be a vital factor affecting the findings. For example, participants with high initial VSWM level may be less affected by short-term exercise intervention and cannot significantly improve their VSWM level, which may lead to bias in results. Moreover, there may also be some objective and artificial differences in the operating procedures of each RCT, such as intervention characteristics, the instruction level and the physical activity practiced. In these included studies, some interventions were purely physical, while others were a combination of physical and cognitive activities. Meanwhile, the difference in the level of instruction from therapists may also lead to the participants receiving the physical activity intervention is not as effective as it should be. Overall, it is difficult to completely unify the results of all studies.

A logical next step for future trials would be to fill the gap in studies on adolescents and middle-aged adults; add studies on young adults, older adults, and men; and report detailed exercise intervention programs, including but not limited to the type of exercise, site and equipment if required, intervention period, intervention frequency, intervention duration, and intervention intensity (reporting criteria for intensity, such as heart rate, subjective feeling, and other ways in which practitioners can timely judge intensity during or after exercise). Second, the improvement effect of ball games on VSWM can also be explored, especially ball games that require quick judgment and decision-making. We have already identified 28 studies; however, the total number of participants enrolled so far (*N* = 1,564) is too small for us to be confident that the treatment is effective. Moreover, about 80% of participants are women. Third, future studies may consider increasing follow-up after intervention, or even strengthening relevant longitudinal studies, to investigate reliable intervention effects in longer time dimensions. This is because it remains unclear how long these changes persist and to what extent they can be preserved with or without continuation of training. Fourth, the quality of RCTs should be improved, and the type of blinding method (i.e., participants blinding, therapist blinding, and assessor blinding) should be appropriately increased to reduce possible bias. More importantly, future research should focus on more specific objectives. Very few studies exist on specific VSWM, and most studies synchronize the measurement and analysis of VSWM with WM, which may lead to bias and ambiguity in the results.

### 4.2. Limitations

Although this meta-analysis used a reasonable and comprehensive analysis method, there are some unavoidable limitations.

1. This meta-analysis investigated the effects of exercise interventions on VSWM capacity. However, because of the few RCTs that have studied VSWM alone and as WM includes VSWM and phonologic WM, the review panel members decided to include WM-related studies and excluded phonologic WM measures after consultation.
2. Different measurement tools may result in various improvements. In the same intervention study, the effect size and significance of the differences could vary greatly if different measures were used (Gentile et al., 2020). This suggests that researchers should be careful in selecting more accurate measurement tools, especially in the measurement of different types of cognitive performance.
3. In the regression analysis model, only two types of exercise classification criteria were included: high-and low-cognitive, and acute and chronic exercise. However, this does not mean that other types of exercise do not have exercise-induced cognitive benefits. Future research should try to combine multiple exercise programs with high exercise-induced cognitive benefits, determine their commonalities, and identify more beneficial types of exercise programs for cognitive improvement.
4. There was a disproportional distribution of studies regarding participants’ age; therefore, we obtained only a few effect sizes for the populations of children, young adults, and seniors. Therefore, the absence of a relationship between age and the summary effect should be interpreted with caution.
5. Regarding participant characteristics, we limited moderator analysis only to age, as this variable was reported in all studies. The sex variable was not reported in one study; therefore, it could not be analyzed as a moderator variable. To derive more personalized exercise recommendations, the influence of other characteristics, such as socioeconomic status and body mass index, needs to be investigated as soon as more studies are available.

## 5. Conclusion

Our meta-regression analysis supports only small to moderate improvements in VSWM of children and seniors after long-term exercise and informs healthy practitioners about ways in which this benefit can be maximized. This meta-analysis confirmed a significant positive impact of physical activity on VSWM in healthy children and seniors; the lack of sufficient statistical power may have accounted for the limited effect observed in young adults. Combining the results of this meta-analysis, prescription of programs involving high-level cognitive engagement, low and moderate exercise intensity, chronic exercise, exercise for >30 min per session, and exercise for more than 3 months is recommended for children and seniors. Practitioners should choose exercise programs based on cost-effective and safety considerations and physical and social accessibility of physical activity sites. Factors such as physical state and weather should also be assessed in advance.

Based on our findings, an ideal exercise program should be recommended for the populations validated as effective in this meta-analysis and systematic review. Improvements in VSWM through physical activity interventions were more significant in healthy children aged 4–11 years and healthy older adults aged 58–88 years than in other age groups. For both groups, we a relatively ideal exercise intervention program would be recommended: (1) low to moderate exercise intensity is suitable. There are two main methods to measure and adjust it, which are 40 to 75 percent of the maximum heart rate and 10 to13 on the Borg Rating of Perceived Exertion (RPE) scale (low intensity exercise is prescribed at 10–11, and moderate intensity at 12–13; Carrie, 2012); (2) three to four sessions per week. More often is not better. It should depend on the state of bodily function; (3) exercise for over 30 min per session; (4) chronic exercise is even better, and exercise regularly for at least more than 3 months; (5) the ideal types of exercise include aerobic exercise (i.e., jogging, brisk walking and aerobics), coordination exercise (i.e., calisthenics, table tennis and badminton) and resistance exercise (i.e., half squat in place, heel in place and kneel push-ups), and team sports are more significant; (6) using motor-cognitive dual-task training could increase the level of cognitive engagement (Norouzi et al., 2019). Incorporate cognitive tasks into exercise routine, such as mental arithmetic, memorizing graphs, and reciting phone numbers backwards. Overall, although this is a universal program, individuals can adjust their exercise plans within this safe range for specific circumstances.

## Data availability statement

The original contributions presented in the study are included in the article/supplementary material, further inquiries can be directed to the corresponding author.

## Author contributions

QZ and YZ: topic, and theoretical basis. QZ, JD, and DL: methodology. QZ, JD, CX, DL, and MY: collecting and sorting out study data. JD and DL: data analysis. QZ and JD: validation. QZ: writing section Introduction, Method, Discussion, and Conclusion. JD: writing section Results. QZ, YZ, and LG: Revision. All authors contributed to the article and approved the submitted version.

## Funding

This study was supported by National Social Science Fund of China (Projects Number: 17BTY090; 18BTY094), and by Basic Research Funds for central universities of Southwest University (Projects Number: SWU1709116, SWU1909322).

## Conflict of interest

The authors declare that the research was conducted in the absence of any commercial or financial relationships that could be construed as a potential conflict of interest.

## Publisher’s note

All claims expressed in this article are solely those of the authors and do not necessarily represent those of their affiliated organizations, or those of the publisher, the editors and the reviewers. Any product that may be evaluated in this article, or claim that may be made by its manufacturer, is not guaranteed or endorsed by the publisher.
